# Lower HDL-cholesterol, a known marker of cardiovascular risk, was associated with depression in type 1 diabetes: a cross sectional study

**DOI:** 10.1186/s12944-019-1009-4

**Published:** 2019-03-18

**Authors:** Eva Olga Melin, Hans Olav Thulesius, Magnus Hillman, Ralph Svensson, Mona Landin-Olsson, Maria Thunander

**Affiliations:** 1 Department of Clinical Sciences, Diabetes Research Laboratory, Lund University, Lund, Sweden; 2 Research and Development, Region Kronoberg, Växjö, Sweden; 3 Primary Care, Region Kronoberg, Växjö, Sweden; 4 Department of Clinical Sciences, Division of Family Medicine, Lund University, Malmö, Sweden; 5 Department of Medicine and Optometry, Linnaeus University, Kalmar, Sweden; 6 Department of Psychology, Linnaeus University, Växjö, Sweden; 7 Department of Endocrinology, Skane University Hospital, Lund, Sweden; 8 Department of Internal Medicine, Central Hospital, Växjö, Sweden

**Keywords:** Type 1 diabetes, Depression, Antidepressants, Serum-lipids, Low-grade inflammation

## Abstract

**Background:**

Depression, metabolic disturbances and inflammation have been linked to cardiovascular disease and mortality. Low levels of high-density lipoprotein cholesterol (HDL-cholesterol), a known marker of cardiovascular risk, have been observed in patients with major depression in psychiatric populations. Our main aim was to explore associations between depression, antidepressants, and metabolic and inflammatory variables in patients with type 1 diabetes (T1D). A secondary aim was to explore variables associated with HDL-cholesterol.

**Methods:**

Cross-sectional design. T1D patients (*n* = 292, men 55%, age18–59 years, diabetes duration ≥1 year) were consecutively recruited from one specialist diabetes clinic. Depression was defined as ≥8 points for Hospital Anxiety and Depression Scale-Depression sub scale. Blood samples, anthropometrics, blood pressure, and data regarding medication and life style were collected from electronic health records. Non-parametric tests, multiple logistic and linear regression analyses were performed.

**Results:**

The depression prevalence was 10 and 8% used antidepressants. Median (q_1_, q_3_) HDL-cholesterol (mmol/l) was for the depressed 1.3 (1.2, 1.5) and for the non-depressed 1.6 (1.3, 1.8), *p* = 0.001. HDL-cholesterol levels (per mmol/l) were negatively associated with depression (Adjusted odds ratio (AOR) 0.2, *p* = 0.007), and the use of antidepressants was positively associated with depression (AOR 8.1, *p* <  0.001). No other metabolic or inflammatory variables, or life style factors, were associated with depression when adjusted for antidepressants. Abdominal obesity was associated with antidepressants in women (AOR 4.6, *p* = 0.029). Decreasing HDL-cholesterol levels were associated with increasing triglyceride levels (*p* <  0.001), increasing high-sensitive C-reactive protein (hs-CRP) levels (*p* = 0.021), younger age (*p* <  0.001), male sex (*p* <  0.001), and depression (*p* = 0.045).

**Conclusions:**

Lower HDL-cholesterol levels, known predictors of cardiovascular disease, were associated with depression in patients with T1D. The use of antidepressants was associated with abdominal obesity in women. Depression, low-grade inflammation measured as hs-CRP, higher triglycerides, male sex, and lower age were independently associated with lower HDL-cholesterol levels.

## Background

Depression is a disease with several somatic consequences both in persons with or without diabetes, such as metabolic changes, disturbances of the corticotropin releasing hormone (CRH) system, immuno-inflammatory changes, and increased cardiovascular and all-cause mortality [1–6]. In diabetes, depression is associated with increased prevalence of all diabetes complications [7]. Depression is, however, a heterogeneous disease where melancholic depression is accompanied by an activated CRH system, anxiety, loss of appetite, weight loss and insomnia, whereas atypical depression is accompanied by CRH deficiency, increased appetite, weight gain, lethargy, and hypersomnia [2]. The metabolic syndrome and inflammatory up-regulations have been linked to atypical depression [1].

LDL-cholesterol is positively, and HDL-cholesterol is inversely associated with cardiovascular disease [8–12]. Previous research support that high levels of LDL-cholesterol are causal in the development of cardiovascular disease [9, 11]. Low levels of HDL-cholesterol are strong predictors of atherosclerosis, cardiovascular disease and mortality [10, 12, 13]. The causal relation between HDL-cholesterol and atherosclerosis is, however, uncertain [10]. A genetic study using mendelian randomisation showed that increased plasma HDL-cholesterol levels did not lower the risk for infarction [8]. In inflammatory states it has been shown that HDL-cholesterol levels are low [14], which is important as inflammatory processes are implicated in cardiovascular disease [15]. High-sensitive C-reactive protein (hs-CRP) is a marker of inflammation that has been shown to predict incident cardiovascular disease and sudden cardiac death [15]. Inadequate glycemic control in type 1 diabetes (T1D) is associated with increased all-cause and cardiovascular mortality [16].

In psychiatric populations, major depression has been linked to lower levels of HDL-cholesterol [1, 17, 18], and low levels of HDL-cholesterol have been observed in persons who had performed suicide attempts [17, 19]. The opposite state, i.e. high levels of psychological well-being, predicted high levels of HDL-cholesterol and low levels of triglycerides in a longitudinal study [20]. HDL-cholesterol is usually high in patients with T1D [21].

In this cohort of patients with T1D we have previously found that depression was associated with anxiety [22], inadequate glycaemic control [23], high midnight cortisol secretion [22, 24], and with galectin-3 [25], an inflammatory biomarker which both predicts heart failure and contributes to cardiac dysfunction [26, 27]. Depression was not associated with abdominal obesity [28]. The increased levels of anxiety, increased midnight cortisol secretion, and lack of obesity in the depressed patients with T1D, indicate traits of melancholic depression [2, 22, 24, 28]. We have also shown that abdominal obesity and high LDL-cholesterol, but not HDL-cholesterol, were associated with cardiovascular complications [29]. Previous analyses in these patients with T1D, showed that the use of antidepressants was associated with abdominal obesity in the women with T1D [28], and was associated with soluble (s) CD163 [30], a biomarker involved in inflammatory processes linked to diabetic retinopathy [31].

We hypothesise that depression and/or the use of antidepressant medication might have impact on metabolic and/or inflammatory variables in patients with T1D. The main aim was to explore associations between depression, antidepressants, and metabolic and inflammatory variables in adult patients with T1D. The secondary aim was to explore variables associated with HDL-cholesterol.

## Methods

### Participants and setting

This study has a cross sectional design and was performed at baseline of a randomized controlled trial (ClinicalTrials.gov: NCT01714986) where Affect School with Script Analysis was tried against Basic Body Awareness Therapy for persons with diabetes, inadequate glycaemic control and psychological symptoms [32]. The patients were consecutively recruited by specialist diabetes physicians or diabetes nurses, at regular follow up visits during the period 03/25/2009 to 12/28/2009 from one secondary care hospital specialist diabetes outpatient clinic, with a catchment population of 125,000 in southern Sweden. Inclusion criteria were T1D, age 18–59 years, and diabetes duration ≥1 year. Exclusion criteria were cancer, hepatic failure, end-stage renal disease, stroke with cognitive deficiency, psychotic disorder, bipolar disorder, severe personality disorder, severe substance abuse, mental retardation or inadequate knowledge of Swedish. Two hundred and ninety-two participants, 67% of the eligible patients, provided written informed consent [23]. A questionnaire was used to assess self-reported depression. Blood samples, anthropometrics and blood pressure (BP) were collected. Data were collected from electronic health records and the Swedish National Diabetes Register (S-NDR) [16].

### Self-reported depression

Self-reported depression was assessed by the Hospital Anxiety and Depression Scale-Depression subscale (HADS-D) which consists of 7 statements, with 4 response alternatives from 0 to 3. Depression was defined as HADS-D ≥ 8 points [33].

### HbA1c and serum lipids

After an overnight fast, blood samples were collected. HbA1c and serum lipids were analysed with a Olympus AU clinical chemistry analyser with high specificity (Olympus AU®, Tokyo, Japan) [34]. The intra-coefficients of variation were for HbA1c < 1.2%; total cholesterol < 2.1%; HDL-cholesterol < 3.0%; LDL-cholesterol < 2,6%; and for triglycerides < 2.2%.

Serum-lipids were measured directly [35]. High HbA1c, defined as > 70 mmol/mol, correspond to the 75th percentile in these patients [23]. The analyses were performed at the department of Clinical Chemistry, Växjö Central Hospital.

### Low-grade inflammation

Hs-CRP samples were collected, centrifugated, and stored at − 70 degrees Celsius until analysed with spectrophotometry on a Roche Cobas C501 instrument at the Diabetes Laboratory, Biomedical Center, Lund University, Lund [29]. The intra-coefficient of variation for the hs-CRP analysis was 1.8–3.3%.

Samples with hs-CRP ≥10 mg/l were excluded as recommended in previous research [15]. Samples stored > 1 year were not included. There were 117 missing hs-CRP measurements. A response analysis was previously performed [29]. It showed that the prevalence of abdominal obesity was lower in the 175 patients with hs-CRP measurements than in the 117 patients with missing hs-CRP measurements. Otherwise they did not differ by medians of age, diabetes duration, systolic blood pressure (SBP) or diastolic blood pressure (DBP), serum-lipids; or by prevalence of high HbA1c (> 70 mmol/mol), antihypertensive drugs (AHD), lipid lowering drugs (LLD), physical inactivity, smoking habits, or cardiovascular complications.

### Anthropometrics and blood pressure

Waist circumference (WC), weight, length and BP were measured according to standard procedures by a nurse. Abdominal obesity was defined as WC ≥1.02 m for men, and as WC ≥0.88 m for women [36]. BMI (kg/m^2^) was calculated and was divided into four weight classes: obesity (≥30), overweight (25 to < 30), normal weight (18.5 to < 25), and underweight (< 18.5) [37].

### Clinical psychiatric diagnoses

Clinical psychiatric diagnoses made prior to recruitment were collected from the medical records and were dichotomized as having or not having a clinical psychiatric diagnosis. Patients with single or recurrent episodes of depression, anxiety, stress related syndromes, and former alcohol substance abuse, but under control at the time for inclusion, were included.

### Episodes of hypoglycemia

A severe episode of hypoglycemia was defined as needing help from another person. Episodes during the last 6 months prior to recruitment were registered.

### Smoking and physical activity

Smokers were defined as having smoked any amount of tobacco during the last year.

Physical activity, which was defined as at least 30 min of moderate activities, was divided into four levels performed weekly: less than once, 1–2 times, 3–5 times, > 5 times, as registered in the S-NDR [16]. Physical activity was also dichotomized into physical inactivity, which was defined as less than 30 min of moderate activities once a week, and physical activity which represents all other levels of physical activity.

### Medication

Antidepressants were SSRIs, SNRIs and/or specific serotonergic antidepressants (N06AB, N06AX16, N06AX11).

LLD were HMG CoA-reductase inhibitors (statins) (C10AA). Indications for LLD were total cholesterol > 4.5 mmol/l (> 1.74 mg/dl) and/or LDL-cholesterol > 2.5 mmol/l (> 97 mg/dl) according to the Swedish national guidelines in 2009 [38].

AHD were calcium antagonists (ATC codes C08CA01–02); angiotensin-converting enzyme (ACE) inhibitors (ATC codes C09AA-BA); angiotensin II antagonists (ATC codes C09CA-DA); diuretics (ATC code C03A); selective beta-adrenoreceptor antagonists (ATC code C07AB). Indications for AHD were SBP > 130 mmHg and/or DBP > 80 mmHg according to the Swedish national guidelines in 2009 [38].

Diabetes specific treatment was divided into two groups: Insulin only (multiple daily insulin injections or continuous subcutaneous insulin infusion), or insulin combined with oral antidiabetic drugs (OAD) (ATC code A10BA02). The indications for OAD prescription in addition to insulin were obesity and insulin resistance.

### Statistical analysis

Analysis of data distribution using histograms revealed that age, diabetes duration, hs-CRP, triglycerides, BMI, and WC were not normally distributed and non-parametric analyses were performed. The Mann-Whitney *U* test was used to compare median values, which were presented as median (quartile (q)_1_, q_3_; min-max). Fisher’s exact test and Linear-by-Linear Association (both two-tailed) were used to analyse categorical data.

Crude odds ratios (CORs) for two dependent variables, depression and the use of antidepressants, were calculated by using simple logistic regression analyses. Variables with *p*-values ≤0.10 for the CORs, and gender and age independent of *p*-value, were entered into multiple logistic regression analyses (Backward: Wald) [23–25, 28–30, 39], with depression and use of antidepressants as dependent variables. These analyses were performed for all and gender specified. The Hosmer and Lemeshow test for goodness-of-fit and Nagelkerke R^2^ were used to evaluate each multiple logistic regression analysis model.

Simple linear regression analyses were performed with HDL-cholesterol as dependent variable. Variables with *p*-values ≤0.10 were entered in multiple linear regression analysis (Backward) [29, 40], with HDL-cholesterol as dependent variable.

Confidence intervals (CIs) of 95% were used. *p* <  0.05 was considered statistically significant. SPSS® version 18 (IBM, Chicago, Illinois, USA) was used for all statistical analyses.

## Results

### Baseline data, comparisons between depressed and non-depressed, and between users and non-users of antidepressants

In this cross-sectional study of patients with T1D (*n* = 292, men 55%, age 18–59 years), 30 depressed patients were compared to 262 non-depressed patients, and 23 users of antidepressants were compared to 269 non-users (Table 1).

**Table 1:** Baseline characteristics for 292 T1D patients, comparisons between depressed and non-depressed, antidepressant users and non-users

		All	Depression (HADS-D ≥ 8p)			Antidepressants		
			Yes	No		Yes	No	
		*N =* 292 ^a^	*N =* 30	*N =* 262	*P* ^b^	*N =* 23	*N =* 269	*P* ^b^
Gender	Men	162 (55)	16 (53)	146 (56)	0.85	11 (48)	151 (56)	0.51
	Women	130 (45)	14 (47)	116 (44)		12 (52)	118 (44)	
Age (years)		(18–59)	48 (38, 53)	42 (31, 50)	0.069 ^c^	49 (34, 54)	42 (31, 50)	0.050 ^c^
Diabetes duration (years)		(1–55)	21 (11, 34)	20 (11, 29)	0.49 ^c^	22 (13, 35)	20 (10, 30)	0.19 ^c^
Depression (HADS-D ≥ 8p)		30 (10)	–	–	–	10 (44)	20 (7)	< 0.001
High HbA1c (> 70 mmol/mol)		80 (27)	14 (47)	66 (25)	0.017	11 (48)	69 (26)	0.029
Total cholesterol (mmol/l)		(2.1–10.9)	4.4 (4.1, 4.9)	4.6 (4.1, 5.2)	0.105 ^c^	4.4 (4.0, 5.2)	4.6 (4.1, 5.2)	0.50 ^c^
Triglycerides (mmol/l)		(0.6–5.9)	0.9 (0.7, 1.6)	0.9 (0.7, 1.2)	0.28 ^c^	1.0 (0.7, 1.7)	0.9 (0.7, 1.2)	0.43 ^c^
HDL-cholesterol (mmol/l)		(0.3–2.7)	1.3 (1.2, 1.5)	1.6 (1.3, 1.8)	0.011 ^c^	1.4 (1.3, 1.8)	1.5 (1.3, 1.8)	0.72 ^c^
LDL-cholesterol (mmol/l)		(0.6–8.3)	2.8 (2.3, 3.2)	2.8 (2.4–3.3)	0.55 ^c^	2.7 (2.1, 3.3)	2.8 (2.4, 3.3)	0.34 ^c^
Hs-CRP ^d^ (mg/l)		(0.3–8.9)	0.9 (0.3, 1.3)	0.6 (0.3, 1.7)	0.53 ^c^	0.9 (0.3, 2.8)	0.7 (0.3, 1.7)	0.70 ^c^
Abdominal obesity ^e^		49 (17)	6 (21)	43 (17)	0.60	8 (35)	41 (16)	0.037
BMI classes ^f^ (kg/m^2^)	(≥30)	35 (12)	5 (17)	30 (12)	0.67 ^g^	5 (22)	30 (11)	0.18 ^g^
	(25 to < 30)	104 (36)	6 (20)	98 (38)		8 (35)	96 (36)	
	(18.5 to < 25)	147 (51)	19 (63)	128 (49)		10 (43)	137 (51)	
	(< 18.5)	4 (1)	0	4 (1)		0	4 (2)	
SBP (mm Hg)		(90–160)	120 (114, 135)	120 (110, 130)	0.93 ^c^	120 (115, 130)	120 (110, 130)	0.95 ^c^
DBP (mm Hg)		(55–100)	70 (69, 78)	70 (70, 75)	0.78 ^c^	75 (70, 80)	70 (68, 75)	0.069 ^c^
Hypoglycemia (severe episodes)		13 (4)	2 (7)	11 (4)	0.63	0	13 (5)	0.61
Smoking ^h^		28 (10)	5 (17)	23 (9)	0.19	1 (4)	27 (11)	0.71
Physical inactivity ^i^ (< 0.5 h/week)		31 (11)	5 (17)	26 (11)	0.35	4 (17)	27 (11)	0.31
Physical activity ^i^	> 5 times/week	99 (36)	12 (41)	87 (35)	0.95 ^g^	8 (35)	91 (36)	0.52 ^g^
	3–5 times/week	86 (31)	8 (28)	78 (32)		9 (39)	77 (30)	
	1–2 times/week	59 (22)	4 (14)	55 (22)		2 (8)	57 (23)	
	< 1 time/week	31 (11)	5 (17)	26 (11)		4 (18)	27 (11)	
Clinical psychiatric diagnosis		41 (14)	16 (53)	25 (10)	< 0.001	23 (100)	18 (7)	< 0.001
Antidepressants		23 (8)	10 (33)	13 (5)	< 0.001	–	–	
LLD ^j^		135 (46)	14 (47)	121 (46)	> 0.99	15 (65)	120 (45)	0.080
AHD ^k^		97 (33)	11 (37)	86 (33)	0.69	10 (44)	87 (32)	0.36
Insulin		275 (94)	29 (97)	246 (94)	> 0.99	21 (91)	254 (94)	0.63
Insulin and OAD ^l^		17 (6)	1 (3)	16 (6)		2 (9)	15 (6)	
Cardiovascular complications		10 (3%)	4 (13)	6 (2)	0.012	5 (22)	5 (2)	< 0.001

Data are n (%), (min-max), or median (q_1_, q_3_). ^a^ Number is 292 unless otherwise specified. ^b^ Fisher’s exact test unless otherwise indicated. ^d^ High - sensitivity C-reactive protein, *n* = 175. ^c^ Mann-Whitney *U* test. ^e^ *N* = 284. ^f^ *N* = 290. ^g^ Linear by linear association. ^h, i^ *N* = 275. ^j^ Lipid lowering drugs. ^k^ Antihypertensive drugs. ^l^ Oral antidiabetic drugs

The median HDL-cholesterol was lower in the depressed than in the non-depressed patients (*p* = 0.011). The prevalence of high HbA1c (> 70 mmol/mol) (*P* = 0.017), use of antidepressants (*p* <  0.001), and cardiovascular complications (*p* = 0.012) were higher in the depressed than in the non-depressed. The prevalence of abdominal obesity (*p* = 0.037), high HbA1c (> 70 mmol/mol) (*p* = 0.029), and cardiovascular complications (*p* < 0.001) were higher in the users of antidepressants.

### Gender sub analyses

In the 16 depressed men median (q_1_, q_3_) HDL-cholesterol (mmol/l) was 1.4 (1.1, 1.6) and in the 146 non-depressed men 1.5 (1.2, 1.7), *p* = 0.20. The prevalence of antidepressants was 5 (31%) in the depressed men, and 6 (4%) in the 146 non-depressed men, *p* = 0.002. Medians did not differ between the depressed and the non-depressed men for total cholesterol, triglycerides, LDL-cholesterol, WC, BMI, hs-CRP, SBP or DBP, all *p*-values ≥0.33. The prevalence did not differ between the depressed and the non-depressed men for high HbA1c, the use of LLD or AHD, all *p*-values ≥0.54. Eleven men used antidepressants. The prevalence of abdominal obesity was in the 11 men using antidepressants 1 (9%) compared to 12 (8%) in the 148 non-users, *p* > 0.99.

In the 14 depressed women median (q_1_, q_3_) HDL-cholesterol (mmol/l) was 1.3 (1.3, 1.6), and in the 116 non-depressed women 1.6 (1.4, 1.9), *p* = 0.008. The prevalence of high HbA1c was 9 (64%) in the depressed women, and 32 (28%) in the non-depressed, *p* = 0.012. The prevalence of use of antidepressants was 5 (36%) in the depressed women, and 7 (6%) in the non-depressed, *p* = 0.004. Medians did not differ between depressed and non-depressed women for total cholesterol, triglycerides, LDL-cholesterol, WC, BMI, hs-CRP, SBP or DBP, all *p*-values ≥0.19. The prevalence did not differ between depressed and non-depressed women regarding the use of LLD or AHD, both *p* -values ≥0.53. The prevalence of abdominal obesity was 7 (58%) in the 12 women using antidepressants, and 29 (25%) in the 115 non-users, *p* = 0.037.

### Associations with depression

HDL-cholesterol levels were negatively associated with depression (per mmol/l) (AOR 0.2, *p* = 0.007) (Table 2).

**Table 2:** Associations with self-reported depression for all 292 T1D patients and gender specifically

	Depression (HADS ≥8p)									
			All *N* = 292				Men *N* = 162		Women *N* = 130	
			Controlled for antidepressants				Controlled for antidepressants			
			No		Yes		Yes		Yes	
	COR	*P*	AOR	*P* ^a^	AOR	*P* ^a^	AOR	*P* ^a^	AOR	*P* ^a^
Gender (women)	1.1 (0.5–2.3)	0.80	1.3 (0.6–3.0)	0.48	1.1 (0.5–2.7)	0.79	–	–	–	–
Age (per year)	1.03 (1.00–1.07)	0.081	1.05 (1.01–1.10)	0.012	1.05 (1.00–1.09)	0.028	1.03 (0.98–1.08)	0.22	–	–
Diabetes duration (per year)	1.01 (0.98–1.04)	0.50	–	–	–	–	–	–	–	–
High HbA1c (> 70 mmol/mol)	2.6 (1.2–5.6)	0.015	2.5 (1.1–5.5)	0.025	2.1 (0.9–4.9)	0.092	–	–	2.9 (0.8–10.2)	0.098
Total cholesterol (per mmol/l)	0.7 (0.5–1.1)	0.16	–	–	–	–	–	–	–	–
Triglycerides (per mmol/l)	1.3 (0.9–1.9)	0.21	–	–	–	–	–	–	–	–
HDL-cholesterol (per mmol/l)	0.2 (0.1–0.8)	0.016	0.2 (0.05–0.6)	0.007	0.2 (0.04–0.6)	0.007	–	–	0.2 (0.03–1.2)	0.083
LDL-cholesterol (per mmol/l)	0.9 (0.5–1.4)	0.55	–	–	–	–	–	–	–	–
Hs-CRP ^c^ (per mg/l)	1.1 (0.8–1.4)	0.49	–	–	–	–	–	–	–	–
Abdominal obesity	1.3 (0.5–3.4)	0.53	–	–	–	–	–	–	–	–
BMI (kg/m^2^) (4 weight classes)	–	0.29	–	–	–	–	–	–	–	–
SBP (per mm Hg)	1.00 (0.97–1.03)	0.95	–	–	–	–	–	–	–	–
DBP (per mm Hg)	1.00 (0.95–1.06)	0.86	–	–	–	–	–	–	–	–
Hypoglycemia (severe episodes)	1.6 (0.3–7.7)	0.54	–	–	–	–	–	–	–	–
Smoking	2.0 (0.7–5.8)	0.19	–	–	–	–	–	–	–	–
Physical inactivity (< 0.5 h/week)	1.8 (0.6–5.0)	0.29	–	–	–	–	–	–	–	–
Physical activity (4 levels)	–	0.52	–	–	–	–	–	–	–	–
Clinical psychiatric diagnosis	10.8 (4.7–24.8)	< 0.001	–	–	–	–	–	–	–	–
Antidepressants	9.6 (3.7–24.6)	< 0.001	–	–	8.1 (3.0–21.9)	< 0.001	10.6 (2.8–40.3)	0.001	5.8 (1.4–23.9)	0.014
LLD	1.02 (0.5–2.2)	0.96	–	–	–	–	–	–	–	–
AHD	1.2 (0.5–2.6)	0.67	–	–	–	–	–	–	–	–
Insulin and OAD	0.5 (0.1–4.1)	0.54	–	–	–	–	–	–	–	–

^a^Multiple logistic regression analysis (Backward: Wald). All (not controlled for antidepressants)/all (controlled for antidepressants)/men/women: Hosmer and Lemeshow Test: 0.912 / 0.376/0.265/0.921; Nagelkerke R Square: 0.121/0.223/0.132/0.236

High HbA1c was associated with depression when the use of antidepressants was not included (AOR 2.5), but not when the use of antidepressants was included (AOR 2.1). Higher age was associated with depression (per year) (AOR 1.05). Depression and the use of antidepressants were associated for both men (AOR 10.6) and women (AOR 5.8) (Table 2).

### Association with the use of antidepressants

In women the association between abdominal obesity and antidepressants was significant (AOR 4.6) (Table 3).

**Table 3:** Associations with antidepressants presented for all 292 T1D patients and gender specifically

Antidepressants								
			All *N* = 292		Men *N* = 162		Women *N* = 127	
	COR	*P*	AOR	*P* ^a^	AOR	*P* ^a^	AOR	*P* ^a^
Gender (women)	1.4 (0.6–3.3)	0.44	1.3 (0.5–3.5)	0.61	–	–	–	–
Age (per year)	1.04 (1.0–1.1)	0.068	1.01 (0.96–1.07)	0.59	1.00 (0.93–1.08)	0.89	1.03 (0.96–1.10)	0.42
Diabetes duration (per year)	1.02 (0.99–1.06)	0.19	–	–	1.04 (0.99–1.10)	0.11	–	–
Depression (HADS ≥8p)	9.6 (3.7–24.6)	< 0.001	10.6 (4.0–28.6)	< 0.001	10.6 (2.8–40.3)	0.001	10.8 (2.4–48.5)	0.002
High HbA1c (> 70 mmol/mol)	2.7 (1.1–6.3)	0.026	1.4 (0.5–3.8)	0.46	–	–	2.8 (0.7–11.1)	0.15
Total cholesterol (per mmol/l)	0.98 (0.6–1.5)	0.92	–	–	–	–	–	–
Triglycerides (per mmol/l)	1.1 (0.7–1.8)	0.72	–	–	–	–	–	–
HDL-cholesterol (per mmol/l)	0.8 (0.3–2.7)	0.76	–	–	–	–	–	–
LDL-cholesterol (per mmol/l)	0.9 (0.5–1.6)	0.81	–	–	–	–	–	–
Hs-CRP ^e^ (per mg/l)	1.1 (0.8–1.5)	0.42	–	–	–	–	–	–
Abdominal obesity ^b^	2.9 (1.2–7.2)	0.024	2.7 (1.0–7.4)	0.052	–	–	4.6 (1.2–18.3)	0.029
BMI (kg/m^2^) (4 weight classes)	–	0.56	–	–	–	–	–	–
SBP (per mm Hg)	1.00 (0.96–1.03)	0.81	–	–	–	–	–	–
DBP (per mm Hg)	1.05 (0.99–1.11)	0.087	1.05 (0.98–1.12)	0.14	–	–	1.08 (0.99–1.18)	0.092
Hypoglycemia	–	> 0.99	–	–	–	–	–	–
Smoking ^c^	0.4 (0.05–3.1)	0.38	–	–	–	–	–	–
Physical inactivity (< 0.5 h/week)	1.8 (0.6–5.5)	0.34	–	–	–	–	–	–
Physical activity (4 levels)	–	0.40	–	–	–	–	–	–
LLD	2.3 (1.0–5.7)	0.063	2.3 (0.9–5.9)	0.099	–	–	–	–
AHD	1.6 (0.7–3.8)	0.28	–	–	–	–	2.3 (0.6–9.2)	0.25
Insulin and OAD	1.6 (0.3–7.5)	0.54	–	–	–	–	–	–

^a^Multiple logistic regression analysis (Backward: Wald). ^b^ N = 292/162/. All/men/women: N = 292/162/127; Hosmer and Lemeshow 0.370/0.787/0.54; Nagelkerke 0.210/0.160/0.274

### Associations with HDL-cholesterol

Depression (*p* = 0.045), triglycerides (*p* = < 0.001) and hs-CRP (*p* = 0.021) were negatively associated with HDL-cholesterol levels, whereas female sex (*p* < 0.001), age (*p* = 0.005) and total cholesterol (*p* < 0.001) were positively associated with HDL-cholesterol levels in 171 patients (Table 4).

**Table 4:** Associations with HDL-cholesterol in 171 patients with T1D

	HDL-cholesterol *N* = 171	
	Unstandardized B coefficients	*P* ^a^
Gender (women)	0.130	0.005
Age (year)	0.008	< 0.001
Diabetes duration (year) ^b^	–	–
Depression (HADS-D ≥ 8p)	−0.146	0.045
High HbA1c (> 70 mmol/mol)	−0.050	0.87
Total cholesterol (mmol/l)	0.144	< 0.001
Triglycerides (mmol/l)	−0.154	< 0.001
LDL-cholesterol ^b^ (mmol/l)	–	–
Hs-CRP ^c^ (mg/l)	−0.034	0.021
Abdominal obesity ^b, d^	–	–
BMI ^e^ (kg/m^2^)	−0.049	0.760
SBP ^b^ (mm Hg)	–	–
DBP ^b^ (mm Hg)	–	–
Hypoglycemia ^b^	–	–
Smoking ^f^	−0.079	0.98
Physical inactivity ^b, f^ (< 0.5 h/week)	–	–
Physical activity ^b, f^ (4 levels)	–	–
Antidepressants ^b^	–	–
LLD ^b^	–	–
AHD ^b^	–	–
Insulin and OAD ^b^	–	–

^a^Multiple linear regression (backward). R Square 0.340. ^b^ *P* > 0.10 in single linear regression. Missing variables: ^c^ *n* = 117; ^d^ *n* = 8; ^e^ *n* = 2; ^f^ *n* = 17

## Discussion

**Fig. 1:**
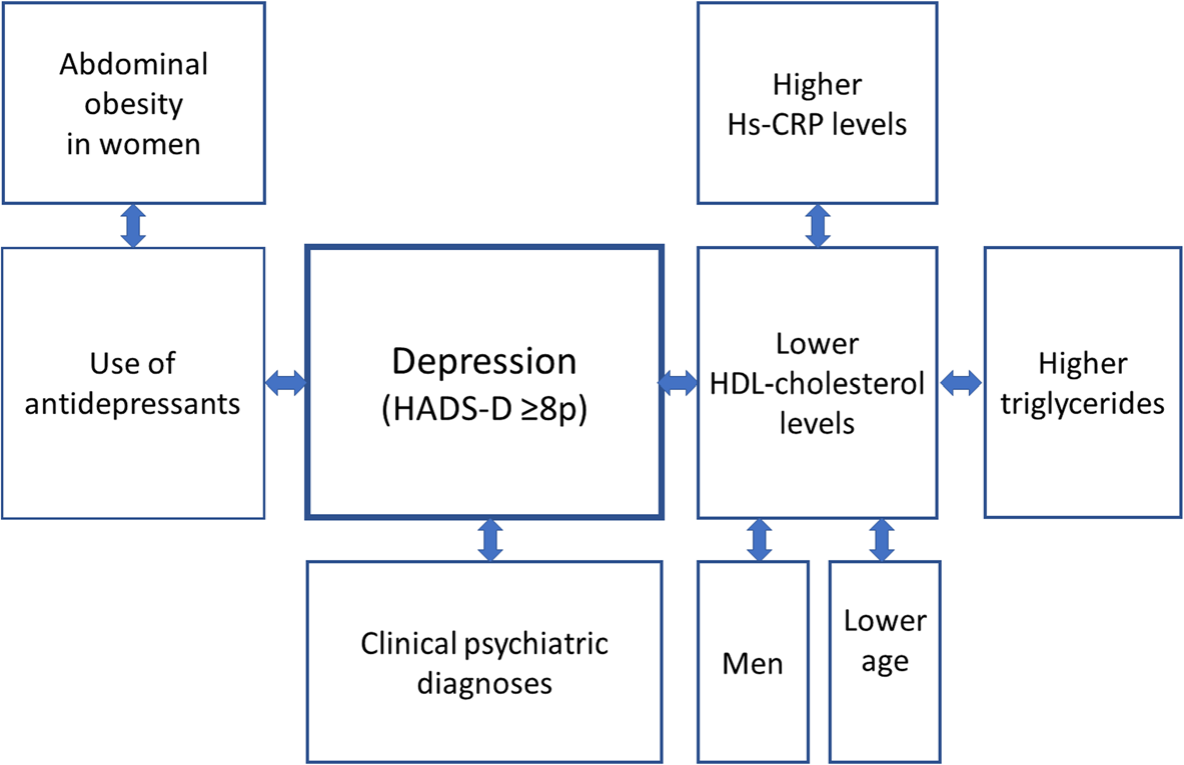
Illustration of significant associations with depression, antidepressants and HDL-cholesterol

The main findings of this study of 292 T1D patients were that self-reported depression was associated with lower HDL-cholesterol levels, and that the use of antidepressants was associated with abdominal obesity in the women (Fig. 1). Depression, low-grade inflammation, higher triglycerides, male sex, and lower age were independently associated with lower HDL-cholesterol levels.

Lower HDL-cholesterol levels were clearly associated with depression in this study. To our knowledge, we are the first to demonstrate an independent association between low HDL-cholesterol and depression in T1D patients. This is intriguing as HDL-cholesterol levels generally are high in patients with T1D [21]. The association between low HDL-cholesterol levels and depression is however in concordance with results in psychiatric populations [1, 17–19]. Lower HDL-cholesterol in depressed patients with T1D may be an important finding as low HDL-cholesterol is a strong predictor of atherosclerosis, cardiovascular disease and mortality [10, 12, 13]. Nevertheless, important evidence from a genetic study using mendelian randomization showed that low HDL-cholesterol levels were not the cause of cardiovascular disease [8]. The depressive state with associated disturbances of the CRF system, metabolic and immuno-inflammatory changes, seems to be an important trigger to increased cardiovascular disease and mortality [16, 26, 27, 41, 42]. HDL-cholesterol levels did not differ between users and non-users of antidepressants medication, which is in accordance with previous research [17]. We found that depression and low-grade inflammation were independently associated with low HDL-levels. Low-grade inflammation has previously been associated with abdominal obesity, cardiovascular disease and mortality [4, 15, 29]. We did not, however, find any association between low-grade inflammation measured as hs-CRP and depression, which is in accordance with previous research where the results were adjusted for BMI [43].

Strengths of the study are that we systematically explored the associations between depression, antidepressant medication, metabolic and inflammatory variables, life style factors, and medication targeting hypertension, dyslipidemia, obesity and insulin resistance. We also systematically explored gender differences. The association between low levels of HDL-cholesterol and depression was strong, despite the limited number of depressed patients. One weakness was that self-reported depression is not equivalent with a clinical diagnosis. We therefore explored the associations between self-reported depression and clinical psychiatric diagnoses, and the use of antidepressants. We found that both these associations were strong. Another weakness was the limited number of patients using antidepressants, particularly when gender sub analyses were performed.

This study does not provide any explanation to why low HDL-cholesterol levels were associated with depression. In previous research we found no association between the depression associated galectin-3 and HDL-cholesterol [25]. With this new finding of lower HDL-cholesterol levels linked to depression in T1D patients, we have altogether demonstrated four biological links to depression, increased midnight cortisol secretion, impaired glycemic control, increased galectin-3 levels, and decreased HDL-cholesterol levels [22–25]. These factors have in in previous research been described as either predictors of risk, or causal in the development of diabetes complications, cardiovascular and all-cause mortality [10, 12, 13, 16, 26, 27, 41, 42].

We suggest in future research of the impact of HDL-cholesterol levels on cardiovascular disease, that depression should be included in the analyses, which has to our knowledge not been done systematically previously. We also suggest comparisons between different types of depression, for example atypical and melancholic depression [2]. It would also be of interest to study whether HDL-cholesterol levels increase after recovery from depression. The mechanisms behind the negative association between depression and HDL is another subject for exploration. We also suggest surveys of this subject in patients with the metabolic syndrome or type 2 diabetes (T2D), and in larger samples of T1D patients.

As inflammatory processes previously have been demonstrated in depression [1, 3, 5, 25], and as HDL-cholesterol levels have recently been shown to decrease with inflammation [14], we plan further exploration of inflammatory markers such as proinflammatory cytokines in patients with T1D and depression [5]. We also advocate comparative research on antidepressants regarding their impact on metabolism, immunology, the CRH system, diabetes complications, and mortality, considering potential gender differences.

## Conclusions

Lower HDL-cholesterol levels, known predictors of cardiovascular disease, were associated with depression in patients with T1D. Abdominal obesity was associated with the use of antidepressants in women. Depression, low-grade inflammation measured as hs-CRP, higher triglycerides, male sex, and lower age were independently associated with lower HDL-cholesterol levels. The detrimental effects previously linked to low HDL-cholesterol levels might be caused by metabolic, endocrinological or immunological changes involved in the depressive state, or in inflammatory disorders.

## Abbreviations

AHD: Antihypertensive drugs
AOR: Adjusted odds ratio
BMI: Body Mass Index
CI: Confidence interval
COR: Crude odds ratio
DBP: Diastolic blood pressure
HADS-D: Hospital Anxiety and Depression-Depression subscale
HDL-cholesterol: High-density lipoprotein cholesterol
Hs-CRP: High-sensitivity C-reactive protein
LDL-cholesterol: Low-density lipoprotein-cholesterol
LLD: Lipid lowering drugs
OAD: Oral antidiabetic drugs
SBP: Systolic blood pressure
S-NDR: Swedish National Diabetes Registry
T1D: Type 1 diabetes
T2D: Type 2 diabetes
WC: Waist circumference
